# Bile micelle binding of structurally diverse ionized drug molecules

**DOI:** 10.5599/admet.2802

**Published:** 2025-07-22

**Authors:** Mayu Konishi, Kiyohiko Sugano

**Affiliations:** Molecular Pharmaceutics Lab., College of Pharmaceutical Sciences, Ritsumeikan University, 1-1-1, Noji-higashi, Kusatsu, Shiga 525-8577, Japan

**Keywords:** Bile micelles, ionizable drug, unbound fraction, dynamic dialysis, intestinal membrane permeation, physiologically-based biopharmaceutics modelling

## Abstract

**Background and purpose:**

Predicting the food effect on oral drug absorption by physiologically based biopharmaceutical modelling (PBBM) remains challenging. The bile micelle unbound fraction (*f*_u_) is one of the primary determinants of the negative food effect for high solubility drugs. To calculate the pH-*f*_u_ profile for PBBM, the bile micelle partition coefficients of ionized and un-ionized drug species (*K*_bm,z_, *z*: charge) are required. The general rules for the ratio of the partition coefficients of ionized and un-ionized drug species have been reported for the octanol/water (*P*_oct_) and phosphatidylcholine liposome/water partition coefficients. However, the general rule for the bile micelle partition coefficient has not yet been investigated. The purpose of the present study was to clarify the general rule for *K*_bm,*z*≠0_/*K*_bm,0_.

**Experimental approach:**

The pH-*f*_u_ profiles of 4 monovalent weak acids, 8 monovalent weak bases, 2 divalent weak bases, and 2 zwitterion drugs were measured by dynamic dialysis in the pH range about p*K*_a_ ± 2. Bile micelles consisted of taurocholic acid (TC)/egg lecithin (15 mM/ 3.75 mM). *K*_bm,*z*_ was calculated from the pH-*f_u_* profiles.

**Key results:**

*K*_bm,-1_/*K*_bm,0_ was ≤ 0.03 for all monovalent acids. *K*_bm,+1_/*K*_bm,0_ ranged from 0.24 to 2.6. *K*_bm,+2_/*K*_bm,0_ was about 0.3. For the two zwitterionic drugs, *K*_bm,-1_/*K*_bm,±0_ was 1.1 and 2.3, and *K*_bm,+1_/*K*_bm,±0_ was 3.9 and 20, respectively. *K*_bm,0_ roughly correlated with *P*_oct_ (r = 0.68).

**Conclusion:**

The bile micelle binding of anionic drug species (*z* = -1) is generally negligible, whereas that of cationic drug species (*z* = +1) can be significant. A general rule for *K*_bm,+1_/*K*_bm,0_ was not found. *K*_bm,+1_/*K*_bm,0_ can be greater than 1 in several cases, suggesting an attractive electrostatic interaction between the positive charge of a drug and the negative charge of TC. These points should be considered in food effect prediction.

## Introduction

Oral drug absorption is affected by various gastrointestinal (GI) conditions [1]. Physiologically based biopharmaceutics modelling (PBBM) is anticipated to be a powerful tool to predict the effect of GI conditions on oral drug absorption. The GI conditions in the fed state differ from those in the fasted state. For example, the concentration of bile micelles (*C*_bm_) increases about 5-fold in the fed state compared to the fasted state [2,3]. It has previously been suggested that the unbound (free) fraction (*f*_u_) of a drug in the intestinal fluid is one of the factors of the negative food effect on the oral absorption of highly soluble weak base drugs [4,5]. An increase in bile micelle binding in the fed state reduces *f*_u_, resulting in a decrease in the effective intestinal permeability (*P*_eff_) and a negative food effect on oral drug absorption (cf. *P*_eff_ is defined based on the total dissolved drug concentration (= bound + unbound concentrations)) [6-10]. Bile micelle-bound drug molecules diffuse across the unstirred water layer (UWL) adjacent to the epithelial membrane; however, only unbound drug molecules permeate the epithelial membrane [5]. When *P*_eff_ is rate-limited by the epithelial membrane permeation, *P*_eff_ ∝ *f*_u_*P*_ep_ (*P*_ep_: the epithelial membrane permeability of unbound drug molecules [5]). The *f_u_* value of an ionizable drug depends on the pH value, which can vary in the small intestine due to factors such as the intestinal position, postprandial conditions, and intra- and inter-individual variations. Therefore, a pH-*f*_u_ profile is required for accurate food effect prediction. To calculate the pH-*f_u_* profile, the bile micelle partition coefficient (*K*_bm,*z*_, *z* = charge) of both un-ionized (*z* = 0) and ionized drug species (*z* ≠ 0) is required. *K*_bm_ is the ratio of drug concentration in the bile micelles to the water phase, normalized by the bile micelle and water concentrations [11].

In the case of the octanol-water partition coefficient (*P*_oct_), the ratios of the partition coefficients of cationic species (*P*_oct,+1_) and anionic species (*P*_oct,-1_) to un-ionized species (*P*_oct,0_) are generally approximated to be about *P*_oct,+1_/ *P*_oct,0_ ≈ 1/1000 and *P*_oct,-1_/ *P*_oct,0_ ≈ 1/10000, respectively (in the presence of 0.15 M NaCl) [12]. In the case of the liposome-water partition coefficient of phosphatidylcholine (PC) liposomes, the ratios of the partition coefficients of cationic species (*K*_PC,+1_) and anionic species (*K*_PC,-1_) to un-ionized species (*K*_PC,0_) are generally approximated to be about *K*_PC,+1_/ *K*_PC,0_ ≈ 1/10 and *K*_PC,-1_/ *K*_PC,0_ ≈ 1/100, respectively [12-14]. However, it has been unknown whether there is such a general approximation rule for the bile micelle partition coefficient. Previously, Schwartz et al. investigated the bile micelle binding of several ionizable drugs by electrokinetic capillary chromatography at pH 7.4 and 10 [15]. It was suggested that only hydrophobic weak base drugs, such as quinine and propranolol, can interact with bile micelles. Castro et al. investigated the *K_bm_* of atenolol, nadolol, and nitrazepam at pH 7.0 and 10.8 by spectrofluorimetry and derivative spectrophotometry [16]. In those studies, the *K*_bm_ of protonated molecular species (*K*_bm,+1_) of atenolol and nadolol was greater than that of un-ionized species (*K*_bm,0_). On the other hand, the *K*_bm_ of the deprotonated molecular species of nitrazepam (mono-anion) (*K*_bm,-1_) was markedly less than *K*_bm,0_. However, the number of drugs was not sufficient to clarify whether there is a general rule for *K*_bm,z≠0_/*K*_bm,0_.

**Figure 1.**
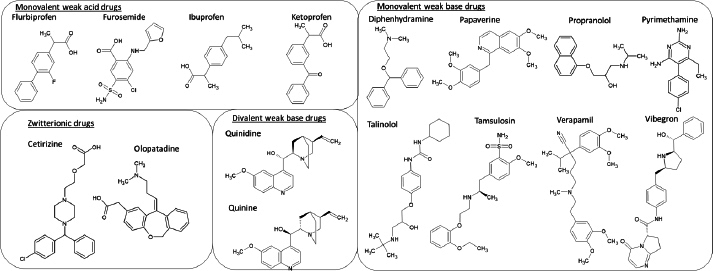
Chemical structures of model drugs

The purpose of the present study was to clarify whether there is a general rule for the ratio of *K*_bm,z≠0_/*K*_bm,0_ for structurally diverse ionizable drug molecules. Four monovalent weak acids, 8 monovalent weak bases, 2 divalent weak bases, and 2 zwitterionic drugs were employed as model drugs (Figure 1). The pH-*f*_u_ profile was measured by dynamic dialysis [17,18]. The *f_u_* value was measured in the pH range of about p*K*_a_ ± 2. The *K*_bm,z_ values were calculated from the pH-*f*_u_ profile in the fed-state simulated intestinal fluid (FeSSIF) containing taurocholic acid (TC) (15 mM) and egg lecithin (EL) (3.75 mM) [19].

**Table 1.** Physicochemical properties of model drugs

Drug	MW	p*K*_a_^a^	log *P*_oct_	ref
Monovalent weak acid drugs				
Flurbiprofen	244	4.18(A) (25 °C, *I* = 0.16 M)	4.0	[12]
Furosemide	331	3.53(A) (37 °C, *I* = 0.15 M)	2.6	[12]
Ibuprofen	206	4.35(A) (25 °C, *I* = 0.15 M)	4.1	[12]
Ketoprofen	254	4.00(A) (37 °C, *I* = 0.15 M)	3.2	[12]
Monovalent weak base drugs				
Diphenhydramine	255	8.86(B) (37 °C, *I* = 0.15 M)	3.2	[12]
Papaverine	339	6.22(B) (37 °C, *I* = 0.18 M)	3.0	[12]
Propranolol	259	9.16(B) (37 °C, *I* = 0.15 M)	3.5	[12]
Pyrimethamine	249	7.36(B) ^b^	2.7	[20]
Talinolol	363	9.4 (B) ^b^	3.1	[21]
Tamsulosin	409	8.37(B) ^b^	2.0	[22]
Verapamil	455	8.68(B) (37°C, *I* = 0.19 M)	4.3	[12]
Vibegron	445	8.9(B) ^b^	3.1	[23]
Divalent weak base drugs				
Quinidine	324	4.09(B), 8.55(B) ^b^	3.6	[12]
Quinine	324	4.35 (B), 8.57 (B) (26°C, *I* = 0.15 M)	3.5	[12]
Zwitterionic drugs				
Cetirizine	389	2.12(B), 2.90(A), 7.98(B) (25°C, *I* = 0.15 M)	1.46 ^c^	[12]
Olopatadine	337	4.18 (A), 9.79 (B) ^b^	0.34 ^c^	[24]

^a^A: acid, B: base

^b^Temperature and ionic strength were not reported

^c^Octanol-water distribution coefficient at pH 6.5 [25].

## Experimental

### Material

Diphenhydramine hydrochloride, ibuprofen, ketoprofen, papaverine hydrochloride, propranolol hydrochloride, quinidine sulphate dihydrate, quinine, sodium taurocholic acid (TC), sodium chloride, sodium dihydrogen phosphate dihydrate, 6 M HCl, and 8 M NaOH were purchased from FUJIFILM Wako Pure Chemical Corporation (Osaka, Japan). Cetirizine dihydrochloride, tamsulosin hydrochloride, pyrimethamine, flurbiprofen, furosemide, olopatadine hydrochloride, talinolol, and verapamil hydrochloride were purchased from Tokyo Chemical Industry Co., Ltd (Tokyo, Japan). Vibegron was extracted from the Beova tablet purchased from KYORIN Pharmaceutical Co., Ltd (Tokyo, Japan). Egg yolk lecithin (EL) was purchased from Kewpie Corporation (Tokyo, Japan). A cellulose dialysis membrane (MWCO 3500) was purchased from As-One Corporation (Osaka, Japan).

### Methods

#### Measurement of the unbound fraction by dynamic dialysis

The *f*_u_ value was measured by dynamic dialysis using a side-by-side chamber (SANPLATEC Co., Ltd, Osaka, Japan) as previously reported [25,26]. The area of the dialysis membrane was 2.0 cm^2^. The fluid volume was 1.5 mL in both the donor and acceptor chambers. The bile micelle media consisted of TC/EL (15 / 3.75 mM) and phosphate buffer (28.6 mM phosphate, 106 mM NaCl). The pH value was adjusted by NaOH in the range of about p*K*_a_±2 (9615S-10D Standard ToupH electrode, HORIBA Advanced Techno, Co., Ltd., Kyoto, Japan). A drug solution with or without bile micelles at each pH (1.5 mL) was added to the donor chamber. The initial donor concentration of each drug is shown in Supplementary material (SM) Table S1. A blank phosphate buffer solution (same pH) without a drug and bile micelles (1.5 mL) was added to the acceptor chamber. After incubation for 1.0 h at 37 °C, the drug concentration in the acceptor chamber was measured by HPLC (Shimazu Prominence LC-20 series, column: ZORBAX Eclipse Plus (C18 2.1×50 mm, 3.5 μm) (Agilent Technologies), flow rate: 0.6 mL min^-1^, column temperature: 40 °C, and injection volume of 10 μL). The mobile phase composition and the detection wavelength are listed in SM Table S2. The determination coefficient of the standard curves was greater than 0.999 in all cases.

Permeation, % was calculated as the ratio of the concentrations in the acceptor chamber at 1.0 h and the theoretical equilibrium concentration in the absence of bile micelles (1/2 of the initial donor concentration). The unbound fraction (*f*_u_) was calculated as the ratio of permeation in the presence and absence of bile micelles.

#### *K*_bm_ calculation

The *K*_bm_ value of each charge species (*K*_bm,z_) was calculated from the pH-*f*_u_ profile [27]. For monovalent weak acid drugs (HA), the un-ionized fraction (*f*_0_) is defined by Equation (1),



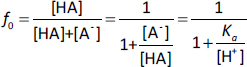

(1)


The unbound fraction (*f*_u_) is defined by Equation (2),



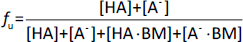

(2)


Therefore, the un-ionized unbound fraction (*f_0_f*_u_) becomes, Equation (3),



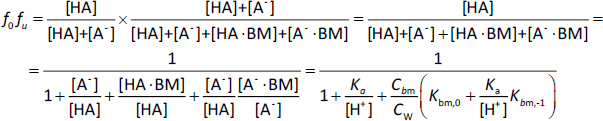

(3)


where BM is bile micelles, *C*_bm_ is the concentration of BM (mol/L) (in this study, 0.015 mol/L), and *C*_W_ is the concentration of water (55.5 mol L^-1^) [11]. The *f*_u_ value can be calculated by dividing Eq. (3) by Eq. (1).

Similarly, for monovalent weak base drugs (B), Equations (4) and (5)



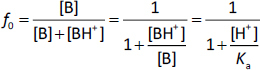

(4)




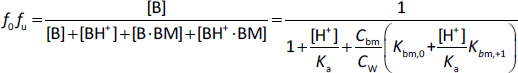

(5)


For divalent weak base drugs with p*K*_a1_ and p*K*_a2_ (B) (p*K*_a1_ < p*K*_a2_), Equations (6) and (7),



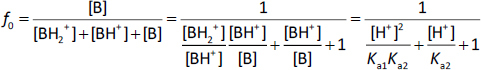

(6)




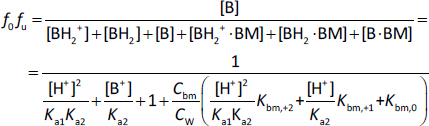

(7)


For zwitterion drugs with p*K_a1_* and p*K_a2_* (D) (p*K_a1_* < p*K_a2_*), Equations (8) and (9),



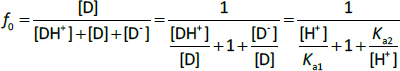

(8)




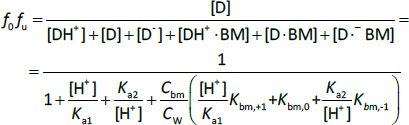

(9)


The *K*_bm,*z*_ values can be obtained by fitting the theoretical pH-*f*_u_ curve to experimentally observed data by the least squares method using the Excel solver. Because the *f*_u_ was measured at 37 °C, the p*K*_a_ value at 37 °C (p*K*_a(37 °C)_) was used for the calculation when available in the literature. The p*K*_a_ value at 25 °C can be converted to p*K*_a(37 °C)_ by the Abraham linear free energy relationship [28]. However, the Abraham solute descriptor was not available for some drugs. In addition, the temperature was not reported. Therefore, in these cases, the reported values were used as it is. [H^+^] was calculated as 10^-pH^, neglecting the effect of ionic strength (*I*) (about 0.1 pH unit) and the electrode factors [12].

## Results and discussion

Permeation and *f*_u_ values at each pH are summarized in SM Table S1. Since the *f*_u_ value becomes sensitive to the variation in permeation at *f*_u_ > 0.9, they were not used for the following data analysis.

### Monovalent weak acid drugs

**Figure 2.**
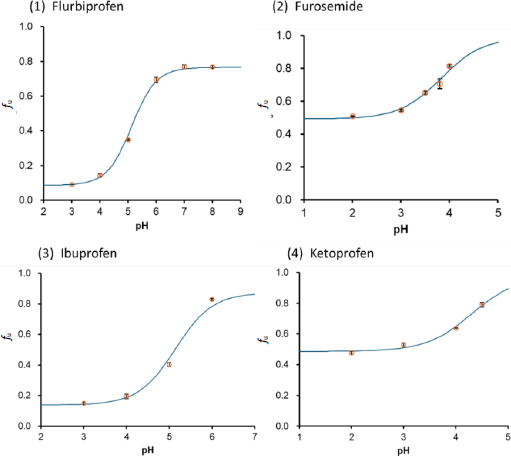
pH-*f*_u_ profile of monovalent weak acids in the TC/EL bile micelle media (mean ± standard deviation (SD), *N* = 3). The solid line is the fitted theoretical curve

Figure 2 shows the pH-*f*_u_ profiles of monovalent weak acid drugs. The theoretical equations (Eqs. (1) to (3)) appropriately described the pH-*f*_u_ profiles. The *K*_bm,*z*_ values are summarized in Table 2.

**Table 2.** *K*_bm_ values of monovalent weak acid drugs in the TC/EL bile micelle media (mean ± SD, *N* = 3)

Drug	*K*_bm,0_	*K*_bm,-1_	*K*_bm,-1_ / *K*_bm,0_
Flurbiprofen	3.93 ± 0.14 × 10^4^	1.14 ± 0.07 × 10^3^	0.03
Furosemide	3.80 ± 0.04 × 10^3^	< 1.00 ×10^2^	<0.03
Ibuprofen	2.29 ± 0.18 × 10^4^	5.33 ± 1.16 × 10^2^	0.02
Ketoprofen	3.91 ± 0.12 × 10^3^	< 1.00 × 10^2^	<0.03

**Figure 3.**
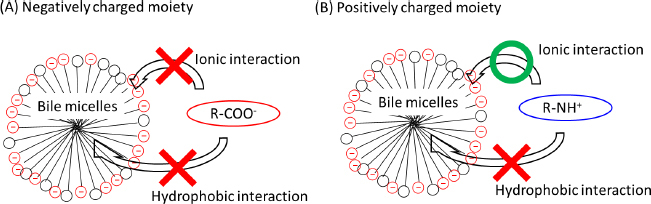
Interactions between the charged moiety of a drug and bile micelles. R-COO^-^: ionized (deprotonated) carboxylic group, R-NH^+^: ionized (protonated) amino group.

In the case of monovalent weak acid drugs, the bile micelle binding decreased as pH increased (the *f*_u_ value increased as a drug became ionized at pH > p*K*_a_ (deprotonated)) (Figure 2). The *K*_bm,-1_/*K*_bm,0_ ratio was ≤ 0.03 for all weak acid drugs (Table 2). Like the cases of *P*_oct_ and *K*_pc_, the bile micelle binding of anionic molecular species (*z* = -1) is negligibly small compared to un-ionized molecular species (*z* = 0). The negatively charged moiety of a drug can be energetically unfavourable for partitioning to both the hydrophobic core region and the negatively charged head group region of the bile micelles (Figure 3A).

### Monovalent weak base drugs

**Figure 4.**
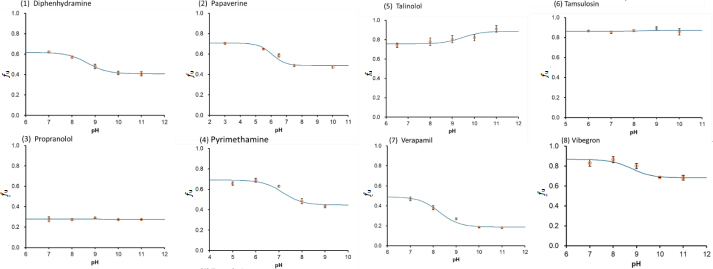
pH-*f*_u_ profile of monovalent weak bases in the TC/EL bile micelle media (mean ± SD, *N* = 3). The solid line is the fitted theoretical curve

Figure 4 shows the pH-*f*_u_ profiles of monovalent weak base drugs. The theoretical equations (Eqs. (4) and (5)) appropriately described the pH-*f*_u_ profiles. The *K*_bm,z_ values are summarized in Table 3.

**Table 3.** *K*_bm_ values of monovalent weak base drugs in the TC/EL bile micelle media (mean ± SD, *N* = 3)

Drug	*K*_bm,0_	*K*_bm, +1_	*K*_bm,+1_/*K*_bm,0_
Diphenhydramine	5.40 ± 0.34 × 10^3^	2.30 ± 0.04 × 10^3^	0.43
Papaverine	3.91 ± 0.16 × 10^3^	1.52 ± 0.03 × 10^3^	0.39
Propranolol	9.66 ± 0.20 × 10^3^	9.64 ± 0.40 × 10^3^	1.00
Pyrimethamine	4.61 ± 0.27 × 10^3^	1.66 ± 0.16 × 10^3^	0.36
Talinolol	4.75 ± 1.00 × 10^2^	1.19 ± 0.11 × 10^3^	2.60
Tamsulosin	5.42 ± 1.29 × 10^2^	5.89 ± 0.39 × 10^2^	1.13
Verapamil	1.60 ± 0.02 × 10^4^	3.87 ± 0.23 × 10^3^	0.24
Vibegron	1.67 ± 0.06 × 10^3^	5.80 ± 1.80 × 10^2^	0.35

In the case of monovalent weak base drugs, the *f*_u_ value either increased or decreased as pH decreased (Figure 3). The *K*_bm,+1_/*K*_bm,0_ ratio ranged from 0.24 to 2.6 (Table 3), unlike the cases of octanol and PC liposome partitioning (*P*_oct,+1_/*P*_oct,0_ ≈ 0.001, *K*_PC,+1_/*K*_PC,0_ ≈ 0.1) [12-14]. Structurally diverse cationic molecular species (*z* = +1) can bind to bile micelles. However, no general rule was found for *K*_bm,+1_/*K*_bm,0_. Therefore, *K*_bm,+1_/*K*_bm,0_ is not simply explained by ionic interaction. The positively charged moiety of a drug can be energetically unfavourable for partitioning to the hydrophobic core region of bile micelles; however, favourable for partitioning to the negatively charged head group region of bile micelles (Figure 3B).

The balance of these two factors can determine the *K*_bm,+1_/*K*_bm,0_ value. In the case of *K*_bm,+1_/*K*_bm,0_ > 1.0, the sulfonate group of taurocholates (R-SO_3_^-^) and the ammonium moiety of a drug (R_3_NH^+^) might have a strong attractive electrostatic interaction [29]. However, in the cases of *K*_bm,+1_/*K*_bm,0_ < 1.0, the cationic charge is less favourable for partitioning into the hydrophobic core region of bile micelles. In the present study, *K*_bm,+1_/*K*_bm,0_ of propranolol and talinolol were ≥ 1.0 (1.0 and 2.6, respectively). Previously, *K_bm,+1_*/*K*_bm,0_ of atenolol and nadolol in deoxycholate/EL micelles were also reported to be > 1.0 (1.3 and 1.7, respectively) [16]. For β-blockers, *K_bm,+1_*/*K_bm,0_* may be generally ≥ 1.0.

### Divalent weak bases

**Figure 5.**
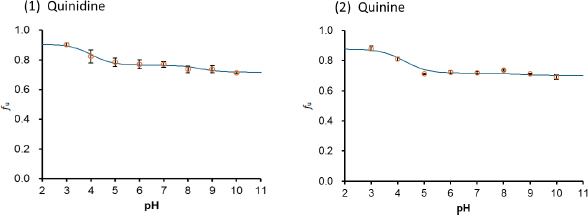
pH-*f*_u_ profile of divalent weak base drugs in the TC/EL bile micelle media (mean ± S.D., N = 3). The solid line is the fitted theoretical curve

Figure 5 shows the pH-*f*_u_ profiles of divalent weak base drugs. The theoretical equations (Eqs. (6) and (7)) appropriately described the pH-*f*_u_ profiles. The *K*_bm,z_ values are summarized in Table 4.

**Table 4.** *K*_bm_ values of divalent base drugs in the TC/EL bile micelle media (mean ± S.D., N = 3)

Drug	*K*_bm,0_	*K*_bm,+1_	*K*_bm,+2_	*K*_bm,+1_/*K*_bm,0_	*K*_bm,+2_/*K*_bm,0_
Quinidine	1.47 ± 0.08 × 10^3^	1.15 ± 0.03 × 10^3^	3.75 ± 1.23 × 10^2^	0.79	0.26
Quinine	1.58 ± 0.07 × 10^3^	1.47 ± 0.02 × 10^3^	5.27 ± 0.43 × 10^2^	0.93	0.34

In the case of the divalent weak base drugs (quinidine and quinine), the bile micelle binding decreased (the *f*_u_ value increased) step by step with decreasing pH below each p*K_a_* (Figure 4). The *K*_bm,+1_/*K*_bm,0_ and *K*_bm,+2_/K_bm,0_ ratios were about 0.8 to 0.9 and 0.3, respectively (Table 4). In these cases, the first and second positive charges are both unfavourable for bile micelle binding. The *K*_bm_ values of quinidine and quinine are almost the same, suggesting that diastereomers may show similar *K*_bm_ values, even though TC and EL are chiral.

### Zwitterionic drugs

**Figure 6.**
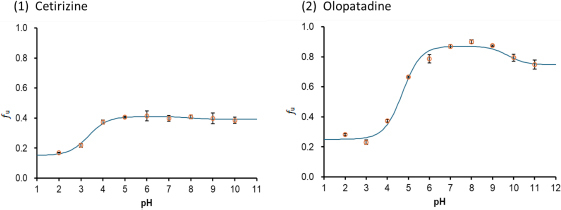
pH-*f*_u_ profile of zwitterionic drugs in the TC/EL bile micelle medium (mean ± SD, *N* = 3). The solid line is the fitted theoretical curve

Figure 6 shows the pH-*f*_u_ profiles of zwitterionic drugs. The theoretical equations (Eqs. (8) and (9)) appropriately described the pH-*f*_u_ profiles. The *K*_bm,z_ values are summarized in Table 5.

**Table 5.** *K_bm_* values of zwitterionic drugs in the TC/EL bile micelle media (mean ± S.D., N = 3)

Drug	*K*_bm,±0_	*K*_bm,-1_	*K*_bm,+1_	*K*_bm,-1_/*K*_bm,±0_	*K*_bm,+1_/*K*_bm,±0_
Cetirizine^a^	5.34 ± 0.33 × 10^3^	5.77 ± 0.61 × 10^3^	2.09 ± 0.10 × 10^4^	1.09	3.93
Olopatadine^a^	5.47 ± 0.38 × 10^2^	1.26 ± 0.15 × 10^3^	1.11 ± 0.04 × 10^4^	2.33	20.5

^a^ See text for the explanation of z = ±0.

In the case of the zwitterionic drugs (cetirizine and olopatadine), the anionic (*z* = -1) and cationic (*z* = +1) species were bound to bile micelles greater than the zwitterionic species (*z* = ±0).

The equilibrium of zwitterionic drugs is expressed by Equation (10),



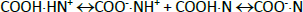

(10)


where COOH is a carboxylic group, and N is an amino group. In the pH region of p*K*_a1_ (acid) < pH < p*K*_a2_ (base), these drugs can exist as un-ionized (*z* = 0, COOH·N) and zwitterionic species (*z* = ±0, COO^-^·NH^+^), the latter being predominant [30,31]. The zwitterionic form contains both negative (*z* = -1) and positive (*z* = +1) charge moieties. In cetirizine and olopatadine, these two moieties are distant from each other and not electrically conjugated. The negatively charged moiety can be energetically unfavourable for partitioning to both the hydrophobic core region and the negatively charged head group region of the bile micelle. On the other hand, the positively charged moiety can be energetically unfavourable for partitioning to the hydrophobic core region; however, favourable for partitioning to the negatively charged head group region. The balance of these factors determines the *K*_bm,z_/ *K*_bm,±0_ value.

*K*_bm,+1_/*K*_bm,±0_ was 3.9 and 20 for cetirizine and olopatadine, respectively (Table 4). These values are greater than the *K*_bm,+1_/*K*_bm,0_ value for monovalent weak base drugs (0.24 to 2.6). When changing pH from pH < p*K*_a1_ to pH > p*K*_a1_, a negative charge is added to the cationic species. (*i.e. z* = +1 + (-1) = ±0). The large *K*_bm,+1_/*K*_bm,±0_ values suggested that the addition of a negative charge is markedly unfavourable for the bile micelle partitioning, like the cases of monovalent weak acids. *K*_bm,+1_/ *K*_bm,±0_ can be rearranged as Equation (11)



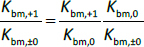

(11)


Therefore, Equation (12)



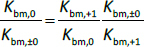

(12)


If the average *K*_bm,+1_/*K*_bm,0_ value for monovalent weak base drugs (0.81) is applied to Eq. 12, *K*_bm,0_/*K*_bm,±0_ becomes 0.21 and 0.041 for cetirizine and olopatadine, respectively. This may suggest that, for bile micelle binding, the zwitterionic species (*z* = ±0, COO^-^-NH^+^) is significantly less favourable than the un-ionized species (*z* = 0, COOH-N). However, a more detailed investigation is required to conclude this point [13].

The *K*_bm,-1_/*K*_bm,±0_ ratios were also greater than 1.0 (1.1 and 2.3 for cetirizine and olopatadine, respectively). The removal of one positive charge from the zwitterion (*i.e. z* = ±0 - (+ 1) = -1) by changing pH from pH < p*K*_a2_ to pH > p*K*_a2_ increased the bile micelle partitioning for these drugs. As discussed above, the removal of a positive charge from a drug can be both favourable and unfavourable for bile micelle partitioning, in this case, favourable.

### Relationship between *K*_bm,0_ and *P*_oct_

**Figure 7.**
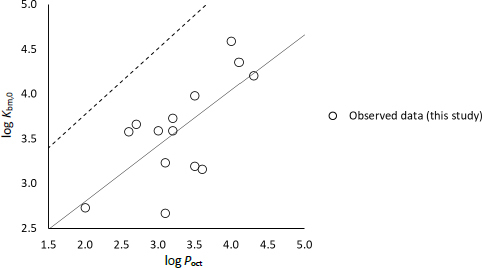
Correlation between log *P*_oct,0_ and log *K*_bm,0_ for TC/EL bile micelles. Solid line: the fitted line in this study, dotted line: the correlation line previously reported by Glomme *et al.* [11]

Previously, Glomme *et al.* [11] reported a good correlation between *K*_bm,0_ and *P*_oct,0_ (log*K*_bm,0_ = 0.74 log*P*_oct,0_ + + 2.29). In that study, *K*_bm,0_ was obtained from the solubility data in bile micelle media for poorly soluble un-ionizable drugs. However, in the present study, only a poor correlation was found between *K*_bm,0_ and *P*_oct,0_ (log*K*_bm,0_ = 0.62 log*P*_oct,0_ + 1.55, *r* = 0.68) (Figure 7). The slope and intercept deviated from the previous report. The reason for this discrepancy is not clear. For high solubility drugs, it is difficult to measure *f*_u_ from the solubility values in bile micelle media. Therefore, dynamic dialysis was used in the present study. This might be one of the reasons for the discrepancy.

### Suggestions for food effect prediction by PBBM

The results of this study suggested that, when predicting the food effect for highly soluble weak acid drugs, *K_bm,-1_* can be negligible. In contrast, for highly soluble weak base drugs, *K*_bm,+1_ should be considered. It was previously reported that the *f*_u_ value at pH 6.5 correlated with the clinical negative food effect for high solubility weak base drugs [4]. At pH 6.5, a weak base drug with p*K_a_* > 7.5 mainly exists as cationic species (> 90%). It was recently reported that quaternary ammonium compounds (permanent cations) can also bind to bile micelles [25,26]. Since there is no general rule for *K*_bm,+1_/*K*_bm,0_, *K*_bm,+1_, and *K*_bm,0_ should be experimentally measured from the pH-*f*_u_ profile. The oral absorption of quinine is reduced by bile micelles in vivo [32], in good agreement with the result of this study. At pH 6.5, quinine mainly exists as z = +1 species. The *f*_u_ values of the zwitterionic drugs (z = ±0) are less than 1 at pH 6.5, in good agreement with the negative food effect [25], suggesting that *K*_bm,±0_ should also be considered. Dynamic dialysis would be a suitable tool to measure *f*_u_ for high solubility drugs [33].

## Conclusions

The bile micelle partitioning of anionic species (*z* = -1) of highly soluble weak acid drugs was negligible. On the other hand, *K*_bm,+1_ /*K*_bm,0_ ranged from 0.24 to 2.6 for highly soluble weak base drugs. In about half of the cases, the mono-cationic species (*z* = +1) were bound to bile micelles equal to or greater than the un-ionized species (*K*_bm,+1_/*K*_bm,0_ ≥ 1.0). Di-cationic (*z* = +2) and zwitterionic species (*z* = ±0) also bound to the bile micelles to some extent. Therefore, the bile micelle binding of *z*=+1, +2 and ±0 species should be considered in food effect prediction.


