# Supplementary material for: Bile micelle binding of structurally diverse ionized drug molecules
Source: ADMET DMPK. 2025 Jul 22;13(4):2802. doi: 10.5599/admet.2802 (PMC12335301; doi:10.5599/admet.2802)
Supplement: Supplementary file 1 [file ADMET-13-2802-S1.pdf]

ADMET & DMPK 13(4) (2025) S2802

Open Access : ISSN : 1848-7718

http://www.pub.iapchem.org/ojs/index.php/admet/index

Supplementary material to

Bile micelle binding of structurally diverse ionized drug molecules

Mayu Konishi and Kiyohiko Sugano*

Molecular Pharmaceutics Lab., College of Pharmaceutical Sciences, Ritsumeikan University, 1-1-1, Noji-higashi, Kusatsu, Shiga 525-8577, Japan

*Corresponding Authors: E-mail: suganok@fc.ritsumei.ac.jp; Tel.: +81-77-561-2773

ADMET & DMPK **13(4)** (2025) 2802; https://doi.org/10.5599/admet.2802

Table S1. Permeation and f_u data

Drug / ionization type / concentration[a]	pH	Permeation, %		f_u
		w/o bile micelles	w/ bile micelles	
Flurbiprofen A 0.016 mM	3	24.0 ± 0.5	2.2 ± 0.0	0.09 ± 0.00
	4	26.1 ± 1.3	3.7 ± 0.3	0.14 ± 0.01
	5	26.4 ± 0.8	9.2 ± 0.1	0.35 ± 0.01
	6	25.9 ± 1.2	18.0 ± 0.4	0.69 ± 0.02
	7	26.2 ± 0.3	20.1 ± 0.3	0.77 ± 0.01
	8	26.7 ± 0.1	20.5 ± 0.3	0.77 ± 0.01
Furosemide A 0.012 mM	2	28.7 ± 0.7	14.7 ± 0.1	0.51 ± 0.01
	3	28.3 ± 0.8	15.4 ± 0.2	0.54 ± 0.01
	3.5	27.5 ± 0.4	17.9 ± 0.2	0.65 ± 0.01
	3.8	27.2 ± 0.9	19.2 ± 0.8	0.71 ± 0.03
	4	27.6 ± 0.9	22.5 ± 0.2	0.82 ± 0.01
Ibuprofen A 0.048 mM	3	26.3 ± 0.5	3.9 ± 0.2	0.15 ± 0.01
	4	22.9 ± 0.7	4.4 ± 0.4	0.19 ± 0.02
	5	25.6 ± 0.3	10.3 ± 0.3	0.40 ± 0.01
	6	24.8 ± 0.5	20.5 ± 0.1	0.83 ± 0.01
Ketoprofen A 0.12 mM	2	32.2 ± 1.2	15.4 ± 0.3	0.48 ± 0.01
	3	24.1 ± 0.2	12.7 ± 0.3	0.53 ± 0.01
	4	19.8 ± 0.2	12.6 ± 0.0	0.64 ± 0.00
	4.5	28.9 ± 1.2	22.8 ± 0.4	0.79 ± 0.02
Diphenhydramine B 0.4 mM	7	26.5 ± 0.5	16.5 ± 0.3	0.62 ± 0.01
	8	26.5 ± 0.8	15.1 ± 0.3	0.57 ± 0.01
	9	23.2 ± 0.3	11.1 ± 0.5	0.48 ± 0.02
	10	23.4 ± 0.3	9.7 ± 0.4	0.42 ± 0.02
	11	23.3 ± 0.6	9.5 ± 0.5	0.41 ± 0.02
Papaverine B 0.016 mM	3	20.4 ± 0.3	14.4 ± 0.2	0.71 ± 0.01
	5.5	21.0 ± 0.6	13.6 ± 0.1	0.65 ± 0.00
	6.5	20.0 ± 0.6	11.7 ± 0.3	0.59 ± 0.01
	7.5	20.1 ± 1.0	9.8 ± 0.1	0.49 ± 0.01
	10	20.0 ± 0.6	9.5 ± 0.2	0.47 ± 0.01
Propranolol B 0.12 mM	7	26.4 ± 0.7	7.3 ± 0.6	0.28 ± 0.02
	8	25.6 ± 0.6	7.0 ± 0.3	0.27 ± 0.01
	9	25.0 ± 0.1	7.3 ± 0.2	0.29 ± 0.00
	10	27.0 ± 1.1	7.4 ± 0.1	0.27 ± 0.00
	11	29.8 ± 0.7	8.2 ± 0.1	0.28 ± 0.00

Drug / ionization type / concentration[a]	pH	Permeation, %		f_u
		w/o bile micelles	w/ bile micelles	
Pyrimethamine<br>B<br>0.008 mM	5	27.2 ± 1.2	17.9 ± 0.5	0.66 ± 0.02
	6	26.8 ± 0.7	18.4 ± 0.5	0.69 ± 0.02
	7	27.4 ± 0.4	17.3 ± 0.3	0.63 ± 0.01
	8	30.1 ± 1.3	14.6 ± 0.7	0.48 ± 0.02
	9	33.2 ± 0.4	14.4 ± 0.5	0.43 ± 0.02
Tamsulosin<br>B<br>0.08 mM	6	21.2 ± 0.9	18.4 ± 0.0	0.87 ± 0.00
	7	21.2 ± 0.7	18.1 ± 0.3	0.85 ± 0.01
	8	20.5 ± 0.6	17.9 ± 0.2	0.87 ± 0.01
	9	20.5 ± 0.6	18.2 ± 0.4	0.89 ± 0.02
	10	20.7 ± 0.5	17.8 ± 0.6	0.86 ± 0.03
Talinolol<br>B<br>0.08 mM	6.5	16.8 ± 0.9	12.5 ± 0.4	0.74 ± 0.02
	8	16.6 ± 0.4	12.9 ± 0.7	0.78 ± 0.04
	9	15.8 ± 0.7	12.7 ± 0.6	0.81 ± 0.04
	10	17.0 ± 0.9	14.0 ± 0.6	0.82 ± 0.03
	11	16.3 ± 0.1	14.9 ± 0.5	0.91 ± 0.03
Verapamil<br>B<br>0.04 mM	7	16.2 ± 0.5	7.6 ± 0.3	0.47 ± 0.02
	8	16.9 ± 0.6	6.4 ± 0.4	0.38 ± 0.02
	9	16.1 ± 0.6	4.4 ± 0.2	0.27 ± 0.01
	10	15.8 ± 0.2	2.9 ± 0.0	0.18 ± 0.00
	11	14.0 ± 0.7	2.5 ± 0.1	0.18 ± 0.01
Vibegron<br>B<br>0.13 mM	7	17.7 ± 0.4	14.7 ± 0.6	0.83 ± 0.03
	8	21.4 ± 0.9	18.5 ± 0.7	0.86 ± 0.03
	9	21.7 ± 0.2	17.3 ± 0.6	0.80 ± 0.03
	10	22.1 ± 0.7	15.2 ± 0.1	0.69 ± 0.00
	11	15.4 ± 0.8	10.5 ± 0.4	0.68 ± 0.02
Cetirizine<br>Z<br>0.5 mM	2	19.9 ± 0.2	3.4 ± 0.0	0.17 ± 0.00
	3	18.5 ± 1.1	4.0 ± 0.2	0.22 ± 0.01
	4	19.2 ± 0.6	7.2 ± 0.3	0.37 ± 0.01
	5	20.2 ± 0.7	8.2 ± 0.1	0.41 ± 0.00
	6	19.3 ± 1.4	8.0 ± 0.6	0.42 ± 0.03
	7	19.9 ± 0.3	7.9 ± 0.4	0.40 ± 0.02
	8	18.6 ± 0.6	7.6 ± 0.2	0.41 ± 0.01
	9	17.2 ± 1.0	6.8 ± 0.6	0.40 ± 0.03
	10	16.4 ± 0.7	6.3 ± 0.3	0.39 ± 0.02
Olopatadine<br>Z<br>0.4 mM	2	20.5 ± 0.3	5.8 ± 0.1	0.28 ± 0.01
	3	21.3 ± 0.2	4.9 ± 0.3	0.23 ± 0.01
	4	20.0 ± 0.3	7.4 ± 0.2	0.37 ± 0.01
	5	19.8 ± 0.3	13.2 ± 0.1	0.67 ± 0.01
	6	21.7 ± 0.9	17.1 ± 0.6	0.79 ± 0.03
	7	20.7 ± 0.3	18.0 ± 0.2	0.87 ± 0.01
	8	18.7 ± 0.2	16.8 ± 0.2	0.90 ± 0.01
	9	19.1 ± 0.4	16.7 ± 0.1	0.87 ± 0.00
	10	19.6 ± 0.3	15.5 ± 0.5	0.79 ± 0.02
	11	17.9 ± 0.7	13.3 ± 0.5	0.75 ± 0.03
Quinidine<br>BB<br>0.04 mM	2	20.7 ± 1.6	18.7 ± 0.9	0.90 ± 0.04
	3	19.2 ± 0.3	17.3 ± 0.2	0.90 ± 0.01
	4	20.6 ± 0.8	16.9 ± 0.9	0.82 ± 0.04
	5	21.2 ± 0.7	16.6 ± 0.6	0.78 ± 0.03
	6	21.4 ± 0.8	16.5 ± 0.6	0.77 ± 0.03
	7	21.6 ± 0.4	16.6 ± 0.4	0.77 ± 0.02
	8	23.8 ± 1.4	17.5 ± 0.5	0.74 ± 0.02
	9	22.8 ± 1.2	16.8 ± 0.6	0.74 ± 0.02
	10	23.1 ± 1.2	16.5 ± 0.1	0.71 ± 0.01

CC BY

Drug / ionization type / concentration[a]	pH	Permeation, %		f_u
		w/o bile micelles	w/ bile micelles	
Quinine BB 0.05 mM	3	20.1 ± 0.4	17.7 ± 0.3	0.88 ± 0.02
	4	21.4 ± 0.3	17.3 ± 0.3	0.81 ± 0.01
	5	22.1 ± 0.3	15.8 ± 0.1	0.71 ± 0.00
	6	21.6 ± 0.2	15.6 ± 0.2	0.72 ± 0.01
	7	22.6 ± 0.4	16.3 ± 0.2	0.72 ± 0.01
	8	22.3 ± 1.0	16.4 ± 0.1	0.74 ± 0.01
	9	22.5 ± 0.7	16.0 ± 0.1	0.71 ± 0.00
	10	21.6 ± 0.3	14.9 ± 0.4	0.69 ± 0.02
Propranolol(GC) B 0.12 mM	7	24.6 ± 0.6	6.9 ± 0.2	0.28 ± 0.01
	8	24.3 ± 0.8	6.7 ± 0.2	0.28 ± 0.01
	9	26.6 ± 0.5	7.0 ± 0.2	0.26 ± 0.01
	10	27.3 ± 1.6	7.3 ± 0.1	0.27 ± 0.00
	11	25.8 ± 0.5	7.4 ± 0.2	0.28 ± 0.01

[a]A: monovalent acid, B: monovalent base, Z: zwitterion, BB: divalent base.

Table S2. HPLC conditions

Drug	UV detection wavelength, nm	Content of acetonitrile, %[a]
Cetirizine	230	38
Diphenhydramine	258	30
Flurbiprofen	248	48
Furosemide	275	40
Ibuprofen	220	50
Ketoprofen	280	45
Olopatadine	300	31
Papaverine	260	25
Propranolol	291	27
Pyrimethamine	273	26
Quinidine	320	15
Quinine	320	15
Talinolol	243	30
Tamsulosin	280	25
Verapamil	230	37
Vibegron	250	20

[a]0.1 % trifluoroacetic acid-acetonitrile / 0.1 % trifluoroacetic acid-water (%)
